# Prophylactic heparin and risk of orotracheal intubation or death in patients with mild or moderate COVID-19 pneumonia

**DOI:** 10.1038/s41598-021-90713-6

**Published:** 2021-05-31

**Authors:** Alessandra Vergori, Patrizia Lorenzini, Alessandro Cozzi-Lepri, Davide Roberto Donno, Gina Gualano, Emanuele Nicastri, Fabio Iacomi, Luisa Marchioni, Paolo Campioni, Vincenzo Schininà, Stefania Cicalini, Chiara Agrati, Maria Rosaria Capobianchi, Enrico Girardi, Giuseppe Ippolito, Francesco Vaia, Nicola Petrosillo, Andrea Antinori, Fabrizio Taglietti, The ReCOVeRI Study Group, Maria Alessandra Abbonizio, Amina Abdeddaim, Elisabetta Agostini, Fabrizio Albarello, Gioia Amadei, Alessandra Amendola, Maria Assunta Antonica, Mario Antonini, Tommaso Ascoli Bartoli, Francesco Baldini, Raffaella Barbaro, Barbara Bartolini, Rita Bellagamba, Martina Benigni, Nazario Bevilacqua, Gianluigi Biava, Michele Bibas, Licia Bordi, Veronica Bordoni, Evangelo Boumis, Marta Branca, Rosanna Buonomo, Donatella Busso, Marta Camici, Flaminia Canichella, Maria Rosaria Capobianchi, Alessandro Capone, Cinzia Caporale, Emanuela Caraffa, Ilaria Caravella, Fabrizio Carletti, Concetta Castilletti, Adriana Cataldo, Stefano Cerilli, Carlotta Cerva, Roberta Chiappini, Pierangelo Chinello, Maria Assunta Cianfarani, Carmine Ciaralli, Claudia Cimaglia, Nicola Cinicola, Veronica Ciotti, Francesca Colavita, Angela Corpolongo, Massimo Cristofaro, Salvatore Curiale, Alessandra D’Abramo, Cristina Dantimi, Alessia De Angelis, Giada De Angelis, Maria Grazia De Palo, Federico De Zottis, Virginia Di Bari, Rachele Di Lorenzo, Federica Di Stefano, Gianpiero D’Offizi, Francesca Evangelista, Francesca Faraglia, Anna Farina, Federica Ferraro, Lorena Fiorentini, Andrea Frustaci, Matteo Fusetti, Marisa Fusto, Vincenzo Galati, Roberta Gagliardini, Paola Gallì, Gabriele Garotto, Ilaria Gaviano, Saba Gebremeskel Tekle, Maria Letizia Giancola, Filippo Giansante, Emanuela Giombini, Guido Granata, Maria Cristina Greci, Elisabetta Grilli, Susanna Grisetti, Marta Iaconi, Giuseppina Iannicelli, Carlo Inversi, Eleonora Lalle, Maria Elena Lamanna, Simone Lanini, Daniele Lapa, Luciana Lepore, Raffaella Libertone, Raffaella Lionetti, Giuseppina Liuzzi, Laura Loiacono, Andrea Lucia, Franco Lufrani, Manuela Macchione, Gaetano Maffongelli, Alessandra Marani, Andrea Mariano, Maria Cristina Marini, Micaela Maritti, Annelisa Mastrobattista, Ilaria Mastrorosa, Giulia Matusali, Valentina Mazzotta, Paola Mencarini, Silvia Meschi, Francesco Messina, Sibiana Micarelli, Giulia Mogavero, Annalisa Mondi, Marzia Montalbano, Chiara Montaldo, Silvia Mosti, Silvia Murachelli, Maria Musso, Michela Nardi, Assunta Navarra, Martina Nocioni, Pasquale Noto, Roberto Noto, Alessandra Oliva, Ilaria Onnis, Sandrine Ottou, Claudia Palazzolo, Emanuele Pallini, Fabrizio Palmieri, Giulio Palombi, Carlo Pareo, Virgilio Passeri, Federico Pelliccioni, Giovanna Penna, Antonella Petrecchia, Ada Petrone, Elisa Pianura, Carmela Pinnetti, Maria Pisciotta, Pierluca Piselli, Silvia Pittalis, Agostina Pontarelli, Costanza Proietti, Vincenzo Puro, Paolo Migliorisi Ramazzini, Alessia Rianda, Gabriele Rinonapoli, Silvia Rosati, Dorotea Rubino, Martina Rueca, Alberto Ruggeri, Alessandra Sacchi, Alessandro Sampaolesi, Francesco Sanasi, Carmen Santagata, Alessandra Scarabello, Silvana Scarcia, Paola Scognamiglio, Laura Scorzolini, Giulia Stazi, Giacomo Strano, Chiara Taibi, Giorgia Taloni, Tetaj Nardi, Roberto Tonnarini, Simone Topino, Martina Tozzi, Francesco Vairo, Maria Beatrice Valli, Laura Vincenzi, Ubaldo Visco-Comandini, Serena Vita, Pietro Vittozzi, Mauro Zaccarelli, Antonella Zanetti, Sara Zito

**Affiliations:** 1 HIV/AIDS Unit, National Institute for Infectious Diseases Lazzaro Spallanzani IRCCS, Via Portuense, 292, 00149 Rome, Italy; 2 Centre for Clinical Research, Epidemiology, Modelling and Evaluation (CREME), Institute for Global Health, UCL, London, UK; 3 Severe and Immune-Depression Associated Infectious Diseases Unit, National Institute for Infectious Diseases Lazzaro Spallanzani IRCCS, Rome, Italy; 4 Respiratory Infectious Diseases Unit, National Institute for Infectious Diseases Lazzaro Spallanzani IRCCS, Rome, Italy; 5 Emerging Infectious Diseases Unit, National Institute for Infectious Diseases Lazzaro Spallanzani IRCCS, Rome, Italy; 6 Hepatology Unit, National Institute for Infectious Diseases Lazzaro Spallanzani IRCCS, Rome, Italy; 7 Intensive Care Unit, National Institute for Infectious Diseases Lazzaro Spallanzani IRCCS, Rome, Italy; 8 Radiology Unit, National Institute for Infectious Diseases Lazzaro Spallanzani IRCCS, Rome, Italy; 9 Cellular Immunology and Pharmacology Laboratory, National Institute for Infectious Diseases Lazzaro Spallanzani IRCCS, Rome, Italy; 10 Laboratory of Virology, National Institute for Infectious Diseases Lazzaro Spallanzani IRCCS, Rome, Italy; 11 Clinical Epidemiology Unit, National Institute for Infectious Diseases Lazzaro Spallanzani IRCCS, Rome, Italy; 12 Scientific Direction, National Institute for Infectious Diseases Lazzaro Spallanzani IRCCS, Rome, Italy; 13 Health Direction, National Institute for Infectious Diseases Lazzaro Spallanzani IRCCS, Rome, Italy; 14 National Institute for Infectious Diseases Lazzaro Spallanzani IRCCS, Rome, Italy

**Keywords:** SARS-CoV-2, Viral infection

## Abstract

Prophylactic low molecular weight heparin (pLMWH) is currently recommended in COVID-19 to reduce the risk of coagulopathy. The aim of this study was to evaluate whether the antinflammatory effects of pLMWH could translate in lower rate of clinical progression in patients with COVID-19 pneumonia. Patients admitted to a COVID-hospital in Rome with SARS-CoV-2 infection and mild/moderate pneumonia were retrospectively evaluated. The primary endpoint was the time from hospital admission to orotracheal intubation/death (OTI/death). A total of 449 patients were included: 39% female, median age 63 (IQR, 50–77) years. The estimated probability of OTI/death for patients receiving pLMWH was: 9.5% (95% CI 3.2–26.4) by day 20 in those not receiving pLMWH vs. 10.4% (6.7–15.9) in those exposed to pLMWH; p-value = 0.144. This risk associated with the use of pLMWH appeared to vary by PaO_2_/FiO_2_ ratio: aHR 1.40 (95% CI 0.51–3.79) for patients with an admission PaO_2_/FiO_2_ ≤ 300 mmHg and 0.27 (0.03–2.18) for those with PaO_2_/FiO_2_ > 300 mmHg; p-value at interaction test 0.16. pLMWH does not seem to reduce the risk of OTI/death mild/moderate COVID-19 pneumonia, especially when respiratory function had already significantly deteriorated. Data from clinical trials comparing the effect of prophylactic vs. therapeutic dosage of LMWH at various stages of COVID-19 disease are needed.

## Introduction

On January 9 2020, the “World Health Organization” (WHO) declared the identification, by Chinese Health authorities, of a novel coronavirus, further classified as severe acute respiratory syndrome coronavirus 2 (SARS-CoV-2)^1^. The outbreak of SARS-CoV-2 was considered to have originally started via a zoonotic transmission associated with the seafood market in Wuhan, China leading to a sharply spreading outbreak of human respiratory disease (COVID-19) in several other countries worldwide. On March 11 2020, WHO declared COVID-19 a pandemic^2^. To date, over 50.7 million COVID-19 cases and 1.2 million deaths have been reported to WHO. Currently, there are more than 3.6 million new cases and over 54 000 new deaths reported^3^.

COVID-19 might be commonly complicated with some hemostatic changes including mild thrombocytopenia^4^ and increased D-dimer levels^5,6^, indicating some forms of coagulopathy^7–10^ that may predispose to thrombotic events, associated with a higher risk of requiring mechanical ventilation, intensive care unit (ICU) admission, or death^6,9,10^. These hemostatic changes are a specific effect of SARS-CoV-2 and a consequence of a cytokine storm that alters the onset of the systemic inflammatory response syndrome as observed in other viral disease^8^. Generally, a correlation between inflammation and coagulation exists: several inflammatory cytokines lead to an impairment of the coagulation pattern, with a consequent imbalance between the procoagulant and anticoagulant states^11^. In the severe acute respiratory syndrome induced by coronavirus, vascular endothelial damage in small and medium sized pulmonary vessels, disseminated intravascular coagulation (DIC), deep venous thrombosis, and pulmonary thromboembolism have been described^12,13^. Hospitalized patients with acute medical illness, including infections such as pneumonia, are at increased risk of thrombotic events^10^ and it is well known that prophylactic anticoagulation reduces that risk^14,15^. Interestingly, heparin and its related derivatives have shown antiviral and anti-inflammatory activities and seem to be beneficial for patients with other diseases^16,17^. As inflammation, atherogenesis, thrombogenesis, and cell proliferation are joint with each other, the pleiotropic effects of heparin and derivatives may have a therapeutic effect and might be relevant in this setting^6,16,18^. Nowadays, physicians treating patients with COVID-19 are facing challenges and one of these is related with the therapeutic utility of heparin^17^. The use of prophylactic-doses of low molecular weight heparin (pLMWH) is now recommended by the WHO^19^ and others guidelines^20–24^ for all hospitalized COVID- 19 patients, unless of clinical contraindications. However, there are conflicting opinions regarding the optimal dose of prophylactic anticoagulation to prevent thrombotic events in COVID-19 patients and to induce a potential anti-inflammatory activity because of the lack of solid evidences.

The aim of this analysis was to assess the effectiveness of prophylactic dose of LMWH vs. no heparin in reducing the risk of orotracheal intubation and death in a real-life setting of patients hospitalized for COVID-19.

## Results

### Patients’ characteristics

A total of 449 patients with COVID-19 mild/moderate pneumonia was included in this analysis. Over 48 h from the date of admission, 210 (46.8%) patients started pLMWH and 239 (53.2%) did not. Overall, 39% were female, with a median (Inter-Quartile Range, IQR) age of 63 (50–77) years and a median of 8 days from onset of symptoms to hospital admission (IQR 4–12).

The main characteristics of the study population at admission, overall and according to pLMWH treatment at admission, are shown in Table 1.

**Table 1:** General characteristics of study population.

	n. 449	n. 239	n. 210	p-value
	Overall	No pLMWH	pLMWH	
**Gender, n (%)**				
Male	275 (61.3)	161 (67.4)	114 (54.3)	0.005
Female	174 (38.8)	78 (32.6)	96 (45.7)	
Age, years, median (IQR)	63 (50–77)	59 (49–71)	72 (55–82)	< 0.001
**Number of co-morbidities, n (%)**				
0	146 (32.5)	107 (44.8)	39 (18.6)	< 0.001
1	109 (24.3)	66 (27.6)	43 (20.5)	
2	73 (16.3)	32 (13.4)	41 (19.5)	
3 +	121 (27.0)	34 (14.2)	87 (41.4)	
PaO_2_/FiO_2_ at admission ≤ 200 mmHg, n (%)	48 (10.7)	18 (7.5)	30 (14.3)	0.013
PaO_2_/FiO_2_ at admission ≤ 300 mmHg, n (%)	133 (29.6)	58 (24.3)	75 (35.7)	0.008
Hyperinflammation at admission^a^, n (%)	260 (57.9)	126 (52.7)	134 (63.8)	0.018
Ferritin, pg/ml, median (IQR)	357 (179–733)	257 (195–767)	357 (163–706)	0.694
C Reactive Protein, mg/dl, median (IQR)	2.7 (1.2–7.0)	2.6 (1.2–6.1)	3.2 (1.3–8.2)	0.205
Lactic dehydrogenase, UI, median (IQR)	233 (185–295)	237 (194–290)	223 (181–300)	0.169
D-dimer, median (IQR)	671 (419–1415)	568 (396–1045)	841 (436–1676)	< 0.001
Lymphocytes, median (IQR)	1220 (860–1720)	1250 (910–1690)	1190 (755–1720)	0.207
**D-dimer, ng/ml, n (%)**				
< 500	141 (31.4)	80 (33.5)	61 (29.1)	< 0.001
501–1000	110 (24.5)	56 (23.4)	54 (25.7)	
1000–2500	92 (20.5)	35 (14.6)	57 (27.1)	
> 2500	42 (9.4)	12 (5)	30 (14.3)	
Missing	64 (14.2)	56 (23.4)	8 (3.8)	
Antiviral therapy started in follow-up, n (%)	364 (81.1)	201 (84.1)	163 (77.6)	0.080
LPV/r	99 (22.1)	71 (29.7)	28 (13.3)	< 0.001
HCQ	91 (20.3)	30 (12.6)	61 (29.1)	
LPV/r + HCQ	169 (37.6)	100 (41.8)	69 (32.9)	
Neither LPV/r nor HCQ	90 (20.0)	38 (15.9)	52 (24.8)	
Remdesivir	16 (3.6)	1 (0.4)	15 (7.1)	< 0.001
Immunomodulant therapy started in follow-up, n (%)	58 (12.9)	22 (9.2)	36 (17.1)	0.012
Steroids, n (%)	157 (35.0)	62 (25.9)	95 (45.2)	< 0.001
Padua score, median (IQR)	1 (0–2)	0 (0–1)	1 (0–2)	< 0.001
Residual normal ventilated lung, volume, L median (IQR)^b^	3.4 (2.4–4.5)	4.0 (2.9–5.1)	3 (2.2–4.2)	0.008

*pLMWH* prophylactic dose of low molecular weight heparin, *IQR* inter quartile range, *COPD* chronic obstructive pulmonary diseases, *LPV/r* lopinavir/ritonavir, *HCQ* hydroxychloroquine.

^a^Defined by the presence of at least two of the following criteria: (a) blood lymphocytes < 1000/mmc; (b) ferritin > 500 ng/mL; (c) LDH > 300 U/L; (d) D-dimers > 1000 ng/mL; (e) C-reactive protein > 3 mg/dL.

^b^Available for 130 patients.

The two groups were considerably different. Patients receiving pLMWH at admission were older, more frequently female and had a higher number of co-morbidities than those who did not receive pLMWH. In the overall study population, we observed 303 (67.5%) patients with more than one comorbidity, a significantly higher proportion of patients with diabetes (23.8% vs 10.5%; p < 0.001), cardiovascular diseases (37.1% vs 18.8%; p < 0.001), hypertension (51.9% vs 29.3%; p < 0.001), COPD/Asthma (25.7% vs 14.2%; p = 0.002), kidney diseases (8.6% vs 2.1%; p = 0.002) and liver disease (8.6% vs 3.4%, p = 0.020) was found among those who received pLMWH at admission versus those who did not. Patients receiving pLMWH had a median PaO_2_/FiO_2_ ratio at admission significantly lower than those not receiving pLMWH [333 mmHg (IQR, 248–400) vs 352 (295–410) respectively; p = 0.05], more frequently met the definition of hyperinflammation condition (64% vs 53%; p = 0.018) and, as expected, showed a higher median d-dimer level [841 ng/mL vs 568, p < 0.001]. The Padua score at admission was higher in the pLMWH group vs. no pLMWH and the volume of normal ventilated lung appeared lower in patients receiving pLMWH than in those who did not [3.0 L (2.2–4.2) vs 4.0 (2.9–5.1); p = 0.008].

**Figure 1:**
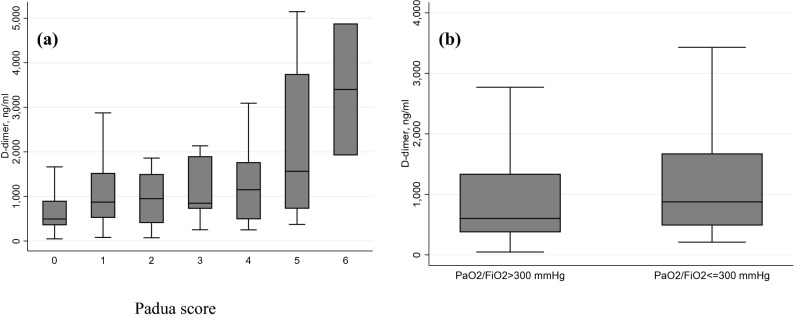
Scatterplot and regression line representing the correlation between **(a)** D-dimer and Padua score and between **(b)** D-dimer and PaO_2_/FiO_2_.

Weak positive correlation was observed for d-dimer level at admission with Padua score (Spearman correlation coefficient =  + 0.28, p < 0.001 and with PaO_2_/FiO_2_ level at admission (Spearman correlation coefficient =  + 0.25, p < 0.001) as showed in Fig. 1.

53% of patients did not receive pLMWH and they were more frequently hospitalized in the first pandemic period (196/239 in March, 33/239 in April and 10 between June and July 2020; p < 0.001).

Overall, only 16 (3.5%) pulmonary thrombosis occurred, of whom 12 were in participants who started pLMWH close to admission and 4 in those who did not. We observed 5 major bleeding events which occurred 4 in people who were treated with heparin and 1 in untreated (p at Fisher exact test 0.076), more in detail: two intramuscular hematomas, 1 cerebral haemorrhage, 1 cerebellar bleeding and a vascular bleeding from the ascending aorta.

As to other treatments, patients on pLMWH received immunomodulant therapy, steroids and remdesivir over follow-up more frequently than patients not treated with pLMWH.

### Primary endpoint OTI/death

**Figure 2:**
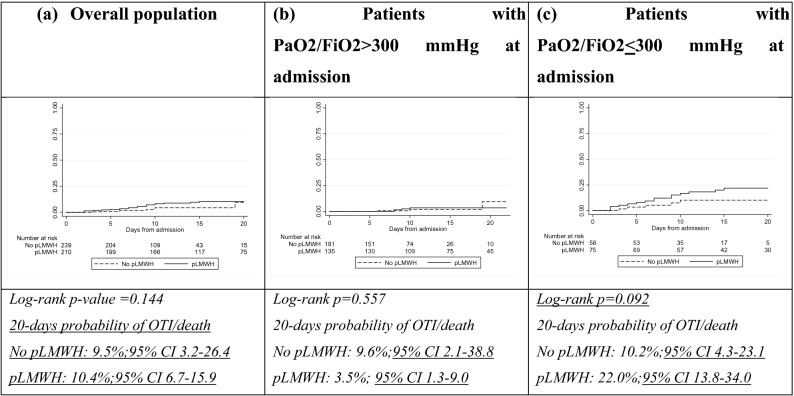
**(a)** Estimated probability of mechanical invasive oro-tracheal intubation/death (OTI/death) according to pLMWH exposure in the study population and stratified by PaO_2_/FiO_2_ ratio at admission **(b)** > 300 mmHg and **(c)** ≤ 300 mmHg. *pLMWH* prophylactic low molecular weight heparin, *OTI/death* oro-tracheal intubation/death.

Over 214 person-months of follow-up, 36 patients experienced OTI or death (6 OTI and 30 death). As expected, the estimated probability of OTI/death was very different according to level of PaO_2_/FiO_2_ at admission (21.3% (95% CI 14.8–30.2) by day 15 in those with PaO_2_/FiO_2_ ratio < 300 mmHg vs. 2.9% (95% CI 1.3–6.3) in those with PaO_2_/FiO_2_ ratio > 300 mmHg; log-rank p-value < 0.001) Fig. 2a. In patients who were hospitalized with PaO_2_/FiO_2_ ≤ 300 mmHg, the probability of OTI/death seemed not different between treatment groups (Fig. 2c), while in patients with PaO_2_/FiO_2_ > 300 at admission, those who did not receive pLMWH showed higher probability of the outcome respect to those who received pLMWH (Fig. 2b).

At multivariable analysis, a first model was adjusted only for time-fixed confounders (model 1) and a second one which included also time-varying confounders concerning concomitant treatment (model 2).

Overall, crude and adjusted marginal hazard ratio for OTI/death showed a reduced risk for patient who received pLMWH but data were highly compatible with the null hypothesis of no difference (model 1: aHR = 0.89, 95% CI 0.34–2.29, p = 0.806; model 2: aHR = 0.66, 95% CI 0.28–1.57, p = 0.352).

After stratifying by baseline PaO_2_/FiO_2_ (> or ≤ 300 mmHg) there was some evidence for a difference in risk by treatment group according to strata. In particular, pLMWH use appeared to be associated with a higher risk of OTI/death among patients admitted with PaO_2_/FiO_2_ ≤ 300 mmHg [model 1 aHR 1.67 (95% CI 0.60–4.67), model 2 aHR 1.40 (95% CI 0.51–3.79)]. In contrast, in the stratum with PaO_2_/FiO_2_ > 300 mmHg, patients receiving pLMWH was consistent with a markedly reduced risk of OTI/death, although with wide confidence intervals [model 1 aHR 0.17 (95% CI 0.01–3.18); model 2 aHR 0.27 (95% CI 0.03–2.18)]. This is indicative of a qualitative interaction although the p-value at interaction test was 0.16 (Table 2).

**Table 2:** Hazard Ratio of oro-tracheal intubation/death (OTI/death) in all population and according to PaO_2_/FiO_2_ at admission.

	Unadjusted and adjusted marginal relative hazards of IOT/death^a^					
	Unadjusted HR (95% CI)	p-value	Adjusted* HR (95% CI)	p-value	Adjusted** HR (95% CI)	p-value
**All patients**						
No pLMWH	1.00		1.00		1.00	
pLMWH	0.85 (0.35, 2.07)	0.727	0.89 (0.34, 2.29)	0.806	0.66 (0.28, 1.57)	0.352
**Baseline PaO**_**2**_**/FiO**_**2**_** ≤ 300 mmHg**						
No pLMWH	1.00		1.00		1.00	
pLMWH	1.68 (0.65, 4.39)	0.287	1.49 (0.52, 4.23)	0.458	1.40 (0.51, 3.79)	
**Baseline PaO2/FiO2 > 300 mmHg**						**Interaction p-value 0.164**
No pLMWH	1.00		1.00		1.00	
pLMWH	0.33 (0.07, 1.48)	0.146	0.25 (0.02, 3.61)	0.310	0.27 (0.03, 2.18)	

*pLMWH* prophylactic dose of low molecular weight heparin.

*Adjusted for time-fixed factors: age, gender, time from symptoms onset, comorbidities (cardiovascular diseases, hypertension, COPD/Asthma, diabetes), PaO_2_/FiO_2_ at admission.

**Adjusted for time-fixed and time varying factors: age, gender, time from symptoms onset, comorbidities (cardiovascular diseases, hypertension, COPD/Asthma, diabetes), PaO_2_/FiO_2_ at admission, time-varying use of immune-therapy, antiviral and steroids and censoring using IPW.

^a^Initiation of invasive mechanical ventilation or death.

The ITT analysis showed similar risk for treated and not treated in the group with PaO_2_/FiO_2_ > 300 and higher risk for treated if the baseline PaO_2_/FiO_2_ was ≤ 300 mmHg (supplementary table 1).

Similar results were obtained after the exclusion of 16 patients with pulmonary thromboembolic events from the study population patients treated with pLMWH showed higher risk of OTI/death versus those not treated if their PaO_2_/FiO_2_ at admission was ≤ 300 mmHg (HR 1.26; 95% CI 0.45–3.53), and they showed a lower risk if they were admitted at hospital with PaO_2_/FiO_2_ > 300 mmHg (HR 0.29; 95% CI 0.04–2.17) (Supplementary table 2).

### Secondary endpoint: death

Over 216 person-months of follow-up, 31 deaths were observed.

At the multivariable analysis on the overall population, we found a signal for a reduced risk of death according to LMWH use [model 1: aHR 0.75 (0.28 to 1.97); p = 0.558; model 2: 0.53 (0.21–1.31); p = 0.168].

Prophylactic LMWH use was associated with a higher, even though not significant, risk of death among patients admitted with a PaO_2_/FiO_2_ ≤ 300 mmHg [model 1: aHR 1.18 (95% CI 0.37–3.79); p = 0.782; model 2: 1.14 (0.37–3.48); p = 0.823], whereas there was some evidence that was a protective factor in the stratum of admission PaO_2_/FiO_2_ > 300 mmHg [model 1: aHR 0.25 (95% CI 0.02–3.59); p = 0.31; model 2: 0.28 (0.03–2.19); p = 0.223] (Table 3).

**Table 3:** Hazard Ratio of death in all population and according to PaO_2_/FiO_2_ at admission.

	Unadjusted and adjusted marginal relative hazards of death					
	Unadjusted HR (95% CI)	p-value	Adjusted* HR (95% CI)	p-value	Adjusted** HR (95% CI)	p-value
**All patients**						
No pLMWH	1.00		1.00		1.00	
pLMWH	0.71 (0.28, 1.80)	0.471	0.75 (0.28, 1.97)	0.558	0.53 (0.21, 1.31)	0.168
**Baseline PaO**_**2**_**/FiO**_**2**_** ≤ 300 mmHg**						
No pLMWH	1.00		1.00		1.00	
pLMWH	1.41 (0.49, 4.01)	0.525	1.18 (0.37, 3.79)	0.782	1.14 (0.37, 3.48)	
**Baseline PaO2/FiO2 > 300 mmHg**						**Interaction p-value 0.216**
No pLMWH	1.00		1.00		1.00	
pLMWH	0.31 (0.07, 1.38)	0.123	0.25 (0.02, 3.59)	0.310	0.28 (0.03, 2.19)	

*Adjusted for time-fixed factors: age, gender, time from symptoms onset, comorbidities (cardiovascular diseases, hypertension, COPD/Asthma, diabetes), PaO_2_/FiO_2_ at admission.

**Adjusted for time-fixed and time varying factors: age, gender, time from symptoms onset, comorbidities (cardiovascular diseases, hypertension, COPD/Asthma, diabetes), PaO_2_/FiO_2_ at admission, time-varying use of immune-therapy, antiviral and steroids and censoring using IPW.

*pLMWH* prophylactic dose of low molecular weight heparin.

## Discussion

This cohort of patients hospitalized for COVID-19 pneumonia at the National Institute for Infectious Diseases L. Spallanzani in Rome, Italy, was mainly enrolled during the first pandemic time-window of the hospitalizations for COVID-19 in Rome. The fact that the evidence was insufficient to determine the risks and benefits of prophylactic anticoagulants for people hospitalized with COVID-19 because of the lack of randomized comparisons on pLMWH versus no treatment and of the availability of few observational studies with no converging results^25^ were the main triggers to perform this analysis.

Patients receiving LMWH prophylaxis (39%) appeared to be older than those who did not receive LMWH prophylaxis, with at least 1 comorbidity; specifically, cardiovascular diseases, hypertension, kidney diseases, COPD/Asthma were the more prevalent.

In our study population, there was a non-negligible proportion of patients, mainly those hospitalized in March/April 2020 (53%) in whom prophylactic LMWH was not prescribed. This finding reflects the fact that in the early stages of the epidemic the risk of thromboembolic events in people with COVID-19 disease had not been clearly recognized. As soon as recommendations were made on prophylactic anticoagulation in COVID-19, since May 2020^19–26^, all hospitalized patients with pneumonia at our COVID-hospital were administered prophylactic dose of LMWH in order to prevent SARS-CoV-2-related thrombotic events. Therefore, this type of analysis will be no longer possible for people enrolled during the second wave of the pandemic.

This analysis reveals that a significant higher proportion of patients receiving LMWH prophylaxis had an impaired respiratory function and a hyperinflammation pattern, which have a known potential prognostic value^5^. These findings highlight that clinicians might have been more prone to use anticoagulant prophylaxis in patients admitted in severe clinical conditions and that respiratory function was the main driver in prescribing LMWH prophylaxis.

Our findings are only partially consistent with those of a meta-analysis showing that adjunctive LMWH use appeared to reduce 7-day and 28-day mortality [RR 0.52 (0.31–0.87 and 0.63 (0.41–0.96), respectively)] as well as improved the PaO_2_/FiO_2_ ratio [by weighted mean difference 74.8 mmHg (52.18–96.78)] in individuals with acute lung injury/acute respiratory distress syndrome (ALI/ARDS) not caused by SARS-Cov-2^27^. The results of this meta-analysis were similar after excluding two studies including more severe patients. Furthermore, our results are also in conflict with those of another observational study in which a better in-hospital survival was shown even in a population with saturation of oxygen < 90% and fever^28^.

More recently, other studies have emerged regarding the risk of mortality in patients treated with heparin such as the experience of the Multicenter Italian CORIST observational study which showed a 40% lower risk of death in patients receiving LMWH or unfractionated heparin [UFH] vs. no heparin (hazard ratio = 0.60; 95% confidence interval: 0.49–0.74; E-value = 2.04) association particularly evident in patients with a higher severity of disease or strong coagulation activation^29^.

The results of a randomized trials, also only recently published^30,31^, add evidence against dose-escalated thromboprophylaxis in critically ill patients with COVID-19. A large observational cohort study of 2,809 critically ill patients with COVID-19 from 67 centers in the US found no benefit of therapeutic dose anticoagulation initiated within 2 days of intensive care unit (ICU) admission compared with standard-dose thromboprophylaxis^32^. Similarly, another Italian study found that the use of a prophylactic dosage of enoxaparin appears to be associated with similar in-hospital overall mortality compared to higher doses in patients hospitalized for COVID-19^33^.

Unfortunately our data do not provide elements to contribute to this debate as we only compared prophylactic dosage with no heparin at all.

Interestingly, in our study the risk of a clinical worsening in patients receiving prophylactic LMWH seemed to vary by the PaO_2_/FiO_2_ ratio at admission. In particular, there appeared to be a qualitative interaction with some evidence that treatment with pLMWH was beneficial in reducing the risk of OTI/death in participants who started the drug with a PaO_2_/FiO_2_ > 300 mmHg but even potentially harmful in those who started with PaO_2_/FiO_2_ ≤ 300 mmHg.

The results for the outcome death alone, were similar to those of the main analysis although with a reduced power to detect the potential interaction with levels of PaO_2_/FiO_2_ and again only partially consistent with those of other observational studies^33–35^.

Regarding the risk of bleeding events, although it was expected, the frequency was very low and there was no difference between pLMWH and no pLMWH.

Our study has some limitations. Firstly, the analysis is retrospective and conducted in the observational setting, therefore residual confounding bias is likely to be an issue. Secondly, this observation includes mainly patients hospitalized in the early stages of the epidemic only in one COVID-hospital in central Italy and may have disproportionately included more patients with better outcomes. Thirdly, only the prophylactic dose of LMWH was evaluated so our data do not contribute to the current debate regarding the identification of the optimal dosage. Last, although an interesting signal was detected regarding a possible role of PaO_2_/FiO_2_ as an effect modifier, the analysis was not powered to detect this interaction.

In conclusion, our results carry little evidence that prophylactic doses of pLMWH can lead to a reduction in risk of OTI/death in patients with mild/moderate COVID-19 pneumonia.

Therefore, overall it seems that prophylactic doses are not sufficient to contrast the hypercoagulable state established in many severe COVID-19 patient, as an obvious consequence of the hyperinflammation and the cytokine storm syndrome and that higher dosage might be needed in people showing generally hyper-inflamed status, impaired respiratory function or suspected high risk of a thrombotic event.

Nevertheless, we have also shown a signal for some clinical benefit of using pLMWH in participants who initiated the drug with a PaO_2_/FiO_2_ > 300 and these data are important to guide future research and the design of randomized studies evaluating the impact of prophylactic heparin vs. higher doses in COVID-19 disease. Our data are compatible with the null hypothesis of no interaction although the effect sizes in the strata are so different that lack of power is a likely explanation for the large p-value. Indeed, the role of prophylactic vs. therapeutic doses LMWH for reducing the risk of thrombosis in hospitalized patients with COVID-19 is currently under evaluation in randomized studies.

## Methods

### Study population

This retrospective analysis included data on patients, ≥ 18 years old, admitted to the National Institute for Infectious Diseases L. Spallanzani in Rome, Italy, with SARS-CoV-2 infection diagnosed by means of RT-PCR positive on naso-pharyngeal swabs (at least once) and/or serology and with a radiologically confirmed mild/moderate pneumonia from 1st March up to 31st July 2020. Data have been collected for the ReCOVeRI Study, a registry on COVID-19 for clinical Research of the National Institute for Infectious Diseases L. Spallanzani, approved by the Ethical Commettee of the National Institute for Infectious Diseases L. Spallanzani IRCCS (number 164, 26 June 2020).

Demographic, epidemiological, clinical data, comorbidities, blood exams, therapeutic data including antibiotic, antiviral and immunomodulating agents (dose, duration and administration mode), oxygen supplementation, were collected and recorded using an electronic database. The management of the registry is adapted according the standards of *EUnetHTA* reported in the Registry Evaluation and Quality Standards Tool (*EUnetHTA, 2019*). All methods were performed in accordance with the relevant guidelines and regulations.

All patients gave informed consent for collecting personal data for research purposes.

CT scans were performed on a multi-detector CT scanner (Bright Speed, GE Medical Systems, Milwaukee, WI). The non-contrast scans were reconstructed with sub-millimetric thicknesses and spacing, high-contrast-resolution algorithm and evaluated to assess the residual pulmonary volume with automatic segmentation of lung areas on dedicated workstation (expressed in Liters).

Patients were included if they were followed-up for at least 2 days after admission. Patients who started a standard prophylactic dose of heparin within 48 h from admission, non-randomly, according to local protocol (intermediate dosage of 100 UI/Kg/day)^25^ were included in the intervention group and compared to the remaining patients who did not receive the drugs.

Patients who started a prophylactic dose of heparin more than 48 h after admission or started a therapeutical dose were excluded from the analysis dataset.

### Definitions

Hyperinflammation condition was defined by the presence of at least two of the following criteria at any time from admission: (a) blood lymphocytes < 1000/mmc; (b) ferritin > 500 ng/mL; (c) LDH > 300 U/L; (d) D-dimers > 1000 ng/mL; (e) C-reactive protein > 3 mg/dL^5^. The Padua score is a tool used to stratify patients and to guide management of the risk of pulmonary embolism^36^.

### Endpoints

The primary endpoint of this analysis was the time to the first event between orotracheal intubation and death (OTI/death). Time to death was analyzed as secondary endpoint.

### Statistical analysis

Patients’ characteristics were described at baseline, non-parametric Mann–Whitney test was used to compare continuous variables and Chi-Square test to compare categorical variables between treatment groups (pLMWH vs. not). Shapiro–Wilk test was used to check for the normality of distribution and the Spearman correlation coefficient was calculated and tested for the correlation analysis. Baseline for the survival analysis was the admission for patients not treated and heparin initiation for treated group. Standard survival analysis by means of weighted Kaplan–Meier (KM) curves were performed to estimate the cumulative proportion of people experiencing the primary endpoint from baseline.

The main analysis was performed using a Cox marginal structural model. The causal HR and corresponding 95% CI of the primary outcome for heparin treated vs not treated participants were estimated by Cox regression model weighted by (i) inverse probability of treatment weights and (ii) censoring weights.

Participants’ follow-up accrued from baseline until the occurrence of the outcome or last in-hospital observation. The follow-up was censored if participants changed the heparin dose from prophylactic to therapeutic. Confounders included for the construction of the weights were: gender, age, duration of symptoms, type of comorbidities, PaO_2_/FiO_2_ measured at admission as time-fixed factors, and the initiation of any antiviral therapy, any immunomodulating agents, any steroids as time-varying factors.

To test the hypothesis of a beneficial effect pLMWH solely via reduction of inflammation, a sensitivity analysis was performed after exclusion of participants who experienced pulmonary thromboembolic events.

The analysis was stratified according to the severity of disease at admission defined as a) PaO_2_/FiO_2_ ratio ≤ or > 300 mmHg. The interaction between PaO_2_/FiO_2_ ratio level and heparin use was formally tested.

The analysis was conducted following both OT and ITT principle, the latter ignored any change in dosage of heparin during observation.

## Supplementary Information

Supplementary Information.

## Data Availability

The datasets generated during and/or analyzed during the current study are available from the corresponding author on reasonable request.
