# Supplementary material for: Prophylactic heparin and risk of orotracheal intubation or death in patients with mild or moderate COVID-19 pneumonia
Source: Sci Rep. 2021 May 31;11:11334. doi: 10.1038/s41598-021-90713-6 (PMC8167179; doi:10.1038/s41598-021-90713-6)
Supplement: Supplementary file 1 — Supplementary Information. [file 41598_2021_90713_MOESM1_ESM.docx]

**Title page**

**Prophylactic heparin and risk of orotracheal intubation or death in patients with mild or moderate COVID-19 pneumonia**

Alessandra Vergori^1^*MD, MD, Patrizia Lorenzini^1^ Stat, Alessandro Cozzi-Lepri^2^ Stat, Davide Roberto Donno^3^, Gina Gualano^4^MD, Emanuele Nicastri^5^MD, Fabio Iacomi^6^MD, Luisa Marchioni^7^MD, Paolo Campioni^8^Prof., Vincenzo Schininà^8^MD, Stefania Cicalini^1^MD, Chiara Agrati^9^MD, Maria Rosaria Capobianchi^10^MD, Enrico Girardi^11^MD, Giuseppe Ippolito^12^Prof., Francesco Vaia^13^MD, Nicola Petrosillo^3^Prof., Andrea Antinori^1^Prof.^¥^, Fabrizio Taglietti^23^MD^¥^, on behalf of the ReCOVeRI Study Group^§^

^1^ HIV/AIDS Unit, National Institute for Infectious Diseases Lazzaro Spallanzani IRCCS, Rome, Italy

^2^Centre for Clinical Research, Epidemiology, Modelling and Evaluation (CREME), Institute for Global Health, UCL, London, UK

^3^Severe and Immune-depression Associated Infectious Diseases Unit, National Institute for Infectious Diseases Lazzaro Spallanzani IRCCS, Rome, Italy

4Respiratory Infectious Diseases Unit, National Institute for Infectious Diseases Lazzaro Spallanzani IRCCS, Rome, Italy

^5^Emerging Infectious Diseases Unit, National Institute for Infectious Diseases Lazzaro Spallanzani IRCCS, Rome, Italy

^6^Hepatology Unit, National Institute for Infectious Diseases Lazzaro Spallanzani IRCCS, Rome, Italy

^7^Intensive care Unit, National Institute for Infectious Diseases Lazzaro Spallanzani IRCCS, Rome, Italy

^8^Radiology Unit, National Institute for Infectious Diseases Lazzaro Spallanzani IRCCS, Rome, Italy

^9^Cellular Immunology and Pharmacology Laboratory, National Institute for Infectious Diseases, Lazzaro Spallanzani IRCCS, Rome, Italy

^10^Laboratory of Virology, National Institute for Infectious Diseases, Lazzaro Spallanzani IRCCS, Rome, Italy

^11^Clinical Epidemiology Unit, National Institute for Infectious Diseases Lazzaro Spallanzani IRCCS, Rome, Italy

^12^Scientific Direction, National Institute for Infectious Diseases Lazzaro Spallanzani IRCCS, Rome, Italy

^13^Health Direction, National Institute for Infectious Diseases Lazzaro Spallanzani IRCCS, Rome, Italy

§ All authors are listed in the Acknowledgments section

¥ These authors contributed equally

***Corresponding Author**

Alessandra Vergori, MD

HIV/AIDS Unit, National Institute for Infectious Diseases L. Spallanzani, IRCCS

Via Portuense, 292, 00149 Rome, Italy

Tel. +390655170420 or +390655170546

e-mail: [alessandra.vergori@inmi.it](mailto:alessandra.vergori@inmi.it)

**Supplementary material**

**Supplementary table 1. ITT analysis: Hazard Ratio of OIT/death in all population and according to PaO2/FiO2 at admission.**

|  | **Unadjusted and adjusted marginal relative hazards of death** | | | |  |  |
| --- | --- | --- | --- | --- | --- | --- |
|  | **Unadjusted**  **HR (95% CI)** | **p-value** | **Adjusted^*^**  **HR (95% CI)** | **p-value** | **Adjusted****  **HR (95% CI)** | **p-value** |
|  | **All patients** | | | |  |  |
| No pLMWH | 1.00 |  | 1.00 |  | 1.00 |  |
| pLMWH | 1.01 (0.46, 2.24) | 0.978 | 1.12 (0.49, 2.57) | 0.785 | 0.82 (0.37, 1.80) | 0.621 |
|  |  | | | |  |  |
|  | **Baseline PaO_2_/FiO_2_ ≤300 mmHg** | | | |  |  |
| No pLMWH | 1.00 |  | 1.00 |  | 1.00 |  |
| pLMWH | 1.48 (0.58, 3.80) | 0.414 | 1.41 (0.51, 3.86) | 0.507 | 1.33 (0.52, 3.39) |  |
|  | **Baseline PaO2/FiO2 >300 mmHg** | | | |  | **Interation p-value**  **0.706** |
| No pLMWH | 1.00 |  | 0.706 |  | 1.00 |  |
| pLMWH | 0.67 (0.18, 2.55) | 0.561 | 0.72 (0.21, 2.45) | 0.600 | 0.95 (0.26, 3.51) |  |
| ^*^adjusted for time-fixed factors: age, gender, time from symptoms onset, comorbidities (cardiovascular diseases, hypertension, COPD/Asthma, diabetes), PaO2/FiO2 at admission | | | | | | |
| ^**^adjusted for time-fixed and time varying factors: age, gender, time from symptoms onset, comorbidities (cardiovascular diseases, hypertension, COPD/Asthma, diabetes), PaO2/FiO2 at admission, time-varying use of immune-therapy, antiviral and steroids and censoring using IPW**Abbreviations**: pLMWH, prophylactic dose of low molecular weight heparin. | | | | | | |

**Supplementary table 2. Hazard Ratio of OIT/death according to PaO2/FiO2 at admission after excluding 16 patients with pulmonary thrombo-embolic events.**

|  | **Unadjusted and adjusted marginal relative hazards of death** | | | |  |  |
| --- | --- | --- | --- | --- | --- | --- |
|  | **Unadjusted**  **HR (95% CI)** | **p-value** | **Adjusted^*^**  **HR (95% CI)** | **p-value** | **Adjusted****  **HR (95% CI)** | **p-value** |
|  | **All patients** | | | |  |  |
| No pLMWH | 1.00 |  | 1.00 |  | 1.00 |  |
| pLMWH | 0.80 (0.33, 1.94) | 0.626 | 0.91 (0.37, 2.22) | 0.835 | 0.60 (0.25, 1.44) | 0.254 |
|  |  | | | |  |  |
|  | **Baseline PaO_2_/FiO_2_ ≤300 mmHg** | | | |  |  |
| No pLMWH | 1.00 |  | 1.00 |  | 1.00 |  |
| pLMWH | 1.53 (0.58, 4.01) | 0.392 | 1.40 (0.49, 4.03) | 0.533 | 1.26 (0.45, 3.53) |  |
|  | **Baseline PaO2/FiO2 >300 mmHg** | | | |  | **Interation p-value**  **0.205** |
| No pLMWH | 1.00 |  | 0.706 |  | 1.00 |  |
| pLMWH | 0.33 (0.07, 1.52) | 0.155 | 0.27 (0.02, 3.92) | 0.338 | 0.29 (0.04, 2.17) |  |
| ^*^adjusted for time-fixed factors: age, gender, time from symptoms onset, comorbidities (cardiovascular diseases, hypertension, COPD/Asthma, diabetes), PaO2/FiO2 at admission | | | | | | |
| ^**^adjusted for time-fixed and time varying factors: age, gender, time from symptoms onset, comorbidities (cardiovascular diseases, hypertension, COPD/Asthma, diabetes), PaO2/FiO2 at admission, time-varying use of immune-therapy, antiviral and steroids and censoring using IPW  **Abbreviations**: pLMWH, prophylactic dose of low molecular weight heparin. | | | | | | |

**Consortium**:

ReCOVeRI Study Group^1^: Maria Alessandra Abbonizio, Amina Abdeddaim, Elisabetta Agostini, Fabrizio Albarello, Gioia Amadei, Alessandra Amendola, Maria Assunta Antonica, Mario Antonini, Tommaso Ascoli Bartoli, Francesco Baldini, Raffaella Barbaro, Barbara Bartolini, Rita Bellagamba, Martina Benigni, Nazario Bevilacqua, Gianluigi Biava, Michele Bibas, Licia Bordi, Veronica Bordoni, Evangelo Boumis, Marta Branca, Rosanna Buonomo, Donatella Busso, Marta Camici, Flaminia Canichella, Maria Rosaria Capobianchi, Alessandro Capone, Cinzia Caporale, Emanuela Caraffa, Ilaria Caravella, Fabrizio Carletti, Concetta Castilletti, Adriana Cataldo, Stefano Cerilli, Carlotta Cerva, Roberta Chiappini, Pierangelo Chinello, Maria Assunta Cianfarani, Carmine Ciaralli, Claudia Cimaglia, Nicola Cinicola, Veronica Ciotti, Francesca Colavita, Angela Corpolongo, Massimo Cristofaro, Salvatore Curiale, Alessandra D’Abramo, Cristina Dantimi, Alessia De Angelis, Giada De Angelis, Maria Grazia De Palo, Federico De Zottis, Virginia Di Bari, Rachele Di Lorenzo, Federica Di Stefano, Gianpiero D’Offizi, Francesca Evangelista, Francesca Faraglia, Anna Farina, Federica Ferraro, Lorena Fiorentini, Andrea Frustaci, Matteo Fusetti, Marisa Fusto, Vincenzo Galati, Roberta Gagliardini, Paola Gallì, Gabriele Garotto, Ilaria Gaviano, Saba Gebremeskel Tekle, Maria Letizia Giancola, Filippo Giansante, Emanuela Giombini, Guido Granata, Maria Cristina Greci, Elisabetta Grilli, Susanna Grisetti, Marta Iaconi, Giuseppina Iannicelli, Carlo Inversi, Eleonora Lalle, Maria Elena Lamanna, Simone Lanini, Daniele Lapa, Luciana Lepore, Raffaella Libertone, Raffaella Lionetti, Giuseppina Liuzzi, Laura Loiacono, Andrea Lucia, Franco Lufrani, Manuela Macchione, Gaetano Maffongelli, Alessandra Marani, Andrea Mariano, Maria Cristina Marini, Micaela Maritti, Annelisa Mastrobattista, Ilaria Mastrorosa, Giulia Matusali, Valentina Mazzotta, Paola Mencarini, Silvia Meschi, Francesco Messina, Sibiana Micarelli, Giulia Mogavero, Annalisa Mondi, Marzia Montalbano, Chiara Montaldo, Silvia Mosti, Silvia Murachelli, Maria Musso, Michela Nardi, Assunta Navarra, Martina Nocioni, Pasquale Noto, Roberto Noto, Alessandra Oliva, Ilaria Onnis, Sandrine Ottou, Claudia Palazzolo, Emanuele Pallini, Fabrizio Palmieri, Giulio Palombi, Carlo Pareo, Virgilio Passeri, Federico Pelliccioni, Giovanna Penna, Antonella Petrecchia, Ada Petrone, Elisa Pianura, Carmela Pinnetti, Maria Pisciotta, Pierluca Piselli, Silvia Pittalis, Agostina Pontarelli, Costanza Proietti, Vincenzo Puro, Paolo Migliorisi Ramazzini, Alessia Rianda, Gabriele Rinonapoli, Silvia Rosati, Dorotea Rubino, Martina Rueca, Alberto Ruggeri, Alessandra Sacchi, Alessandro Sampaolesi, Francesco Sanasi, Carmen Santagata, Alessandra Scarabello, Silvana Scarcia, Paola Scognamiglio, Laura Scorzolini, Giulia Stazi, Giacomo Strano, Chiara Taibi, Giorgia Taloni, Tetaj Nardi, Roberto Tonnarini, Simone Topino, Martina Tozzi, Francesco Vairo, Maria Beatrice Valli, Laura Vincenzi, Ubaldo Visco-Comandini, Serena Vita, Pietro Vittozzi, Mauro Zaccarelli, Antonella Zanetti and Sara Zito

^1^National Institute for Infectious Diseases L. Spallanzani, IRCCS

**Consortia Representative**: Dr Pierluca Piselli, Clinical Epidemiology Unit, National Institute for Infectious Diseases Lazzaro Spallanzani IRCCS, Rome, Italy
